# Volume-Outcome Relation for Acute Appendicitis: Evidence from a Nationwide Population-Based Study

**DOI:** 10.1371/journal.pone.0052539

**Published:** 2012-12-26

**Authors:** Po-Li Wei, Shih-Ping Liu, Joseph J. Keller, Herng-Ching Lin

**Affiliations:** 1 Division of General Surgery, Department of Surgery, Taipei Medical University Hospital, Taipei, Taiwan; 2 Department of Surgery, School of Medicine, College of Medicine, Taipei Medical University, Taipei, Taiwan; 3 Department of Urology, National Taiwan University Hospital and College of Medicine, Taipei, Taiwan; 4 School of Public Health, Taipei Medical University, Taipei, Taiwan; 5 School of Health Care Administration, Taipei Medical University, Taipei, Taiwan; D’or Institute of Research and Education, Brazil

## Abstract

**Background:**

Although procedures like appendectomy have been studied extensively, the relative importance of each surgeon's surgical volume-to-ruptured appendicitis has not been explored. The purpose of this study was to investigate the rate of ruptured appendicitis by surgeon-volume groups as a measure of quality of care for appendicitis by using a nationwide population-based dataset.

**Methods:**

We identified 65,339 first-time hospitalizations with a discharge diagnosis of acute appendicitis (International Classification of Disease, Ninth Revision, Clinical Modification (ICD-9-CM) codes 540, 540.0, 540.1 and 540.9) between January 2007 and December 2009. We used “whether or not a patient had a perforated appendicitis” as the outcome measure. A conditional (fixed-effect) logistic regression model was performed to explore the odds of perforated appendicitis among surgeon case volume groups.

**Results:**

Patients treated by low-volume surgeons had significantly higher morbidity rates than those treated by high-volume (28.1% vs. 26.15, p<0.001) and very-high-volume surgeons (28.1% vs. 21.4%, p<0.001). After adjusting for surgeon practice location, and teaching status of practice hospital, and patient age, gender, and Charlson Comorbidity Index, and hospital acute appendicitis volume, patients treated by low-volume surgeons had significantly higher rates of perforated appendicitis than those treated by medium-volume surgeons (OR = 1.09, p<0.001), high-volume surgeons (OR = 1.16, p<0.001), or very-high-volume surgeons (OR = 1.54, p<0.001).

**Conclusion:**

Our study suggested that surgeon volume is an important factor with regard to the rate of ruptured appendicitis.

## Introduction

Acute appendicitis is the most common surgical emergency. Although the exact mechanism is still obscure, it is likely that luminal obstruction plays a key pathogenic role [1]–[3]. Luminal obstruction leads to increased mucus production and bacterial overgrowth, which increases the intra-luminal pressure and decreases blood ﬂow to the appendiceal wall. Subsequently, necrosis and perforation of the appendix occur. The primary adverse outcome of appendicitis is appendiceal rupture. In comparison with simple appendicitis, patients with an appendiceal rupture at the time of surgical exploration have been shown to have a higher risk of post-operative complication, such as intra-abdominal abscess, wound infection, and postoperative paralytic ileus (39% vs 8%) [4], [5]. It has been assumed that the natural history of appendiceal rupture is within the control of the hospital and attending physician, and that a high rate of rupture reflects a failure of medical care [6]. As a result, appendiceal rupture rates have been proposed as a measure of intrinsic hospital quality, and early surgical exploration has been encouraged to decrease the rupture rate [7], [8].

The impact of hospital volume on operative mortality with surgical procedures has been previously studied [9]. One recent review suggested that thousands of preventable surgical morbidities and mortalities occur each year in the United States, and that patients can significantly reduce their risk of operative death by selecting a high-volume hospital [10]. Despite recent interest in surgical volume, many question the applicability of previous research to the volume and outcomes of current practice.

First, due to recent decreases in surgical mortality associated with many procedures [11], it is possible that the relative importance of the volume of procedures performed may be declining. Second, most published studies on volume and outcome have used state-level databases or regional populations that are served by a small number of high-volume centers [12], [13].

Although procedures like appendectomy have been studied extensively, the relative importance of each surgeon's surgical volume to ruptured appendicitis has either not been explored or has been studied in samples that were too small to permit assessment of performance at meaningful surgical volumes. The purpose of this study was to investigate the rate of ruptured appendicitis with surgeon volume groups as a measure of quality of care for appendicitis by using a nationwide, population-based dataset (from 2007 through 2009). Since some previous studies have suggested that volume-outcome relationships may exist for surgeons but not for hospitals in Taiwan [14], [15], the present study focused on the volume-outcome relationship for surgeons. However, we took hospital volume into consideration in the regression model.

## Methods

### Database

We used data sourced between 2007 and 2009 from the National Health Insurance Research Database (NHIRD). The NHIRD, published by the Taiwan National Health Research Institute, includes all the original claims data and registration files for beneficiaries enrolled under the National Health Insurance (NHI) program. As the end of 2009, about 22.60 of Taiwan’s 22.96 million residents (over 98% coverage rate) were enrolled under the NHI program. Taiwan’s NHI program is a single plan with generous benefits, very low co-payments, and free choice of a widely-dispersed network of healthcare providers. Furthermore, the NHI Bureau implements routine sample cross-checks between each hospital’s claims and medical charts, followed by punitive measures for coding infractions to deter diagnosis upcoding. Therefore, although there are no documented sensitivity and specificity studies for coding accuracy, it is generally believed that the NHI’s checks and balances foster accurate coding.

This study was exempted from full review by an Institutional Review Board (IRB) after consulting with the director of the Taipei Medical University IRB since the NHIRD consists of de-identified secondary data released to the public for research purposes.

### Study Sample

We identified 65,339 first-time hospitalizations with a discharge diagnosis of acute appendicitis (International Classification of Disease, Ninth Revision, Clinical Modification (ICD-9-CM) codes 540, 540.0, 540.1 and 540.9) between January 2007 and December 2009. If a patient had ≥2 hospitalizations within a 30-day period, they were regarded as the same episode and we only included the first hospitalization. The mean age for these 65,339 patients was 36.7 (±19.3 years) years, with a range between 2 and 97 years.

### Surgeon Acute Appendicitis Volume Groups

The NHIRD included unique physician identifiers for each medical claim submitted, which enabled us to identify the same physician who performed one or more appendectomies during the three-year study period. Surgeons were sorted in ascending order of appendectomy volume, with volume cutoff points being determined in accordance with prior studies so that the sampled patients were classified into four, approximately equally-sized groups [6], [7]. We divided the sample of 65,339 patients into four surgeon volume groups: ≤65 cases (hereafter referred to as low volume), 66–120 cases (medium volume), 121–190 (high volume), and ≥191 cases (very-high volume).

### Key Variables of Interest

The key independent variable of interest was surgeon appendectomy caseload volume. Prior studies have used the proportion of cases of perforated appendicitis as a measure of quality of care for appendicitis [16], [17]. Therefore, in this study, we used “whether or not a patient had a perforated appendicitis” as the outcome measure. Patients were regarded as having perforated appendicitis if they had an ICD-9-CM code of 540.0 (acute appendicitis with generalized peritonitis) or 540.1 (acute appendicitis with peritoneal abscess).

### Statistical Analysis

We used the SAS package (Version 9.1) for statistical analysis in this study. Chi-squared tests were used to examine differences among surgeon volume groups in terms of both surgeon and patient characteristics. Surgeon characteristics included age, gender, practice location (urban vs. rural), and teaching status of practice hospital, while patient characteristics included age, gender, and Charlson Comorbidity Index (CCI). CCI was used to quantify pre-existing co-morbidities as a means of adjusting for the higher mortality risks associated with co-morbidities (the higher the score, the greater the number of co-morbidities). We then performed a conditional (fixed-effect) logistic regression model (conditional on hospitals in order to partition out systematic hospital-specific variation) to explore the odds of perforated appendicitis among surgeon case volume groups. This conditional model also uses a clustered method for variance estimation to account for the possibility that the patients of each physician have more similar outcomes than patients viewed across physicians. A two-sided *p* value of less than, or equal to, 0.05 was considered to be statistically significant.

## Results

Table 1 presents the distribution of the rate of perforated appendicitis and patient and surgeon characteristics across surgeon acute appendicitis case volume groups. Of the 65,339 patients admitted for the treatment of acute appendicitis between January 2007 and December 2009, 16,857 (25.8%) had perforated appendicitis; the rate of perforated appendicitis consistently decreased with increasing surgeon acute appendicitis caseload volume. Patients treated by low-volume surgeons had significantly higher morbidity rates than those treated by high-volume (28.1% vs. 26.15, *p*<0.001) or very-high-volume surgeons (28.1% vs. 21.4%, *p*<0.001). In addition, patients in the low surgeon volume quartile were more likely to have a greater number of co-morbidities (*p*<0.001).

**Table 1:** Perforated appendicitis rate and patient and physician characteristics across physician pulmonary embolism caseload volume groups (n = 65,339).

Variable	All	Physician acute appendicitis case volume				*p* value
		Low(≤65)	Medium(66∼120)	High(121∼190)	Very High(≥191)	
Perforated appendicitis rate (%)	25.8	28.1	27.0	26.1	21.4	<0.001
**Patient characteristics**						
No. (%) of patients	65,339	16,094 (24.6)	16,614 (25.4)	18,055 (27.7)	14,576 (22.3)	
Age, mean (SD), years	36.7 (19.3)	36.5 (21.0)	37.5 (19.4)	37.2 (18.3)	35.5 (18.4)	<0.001
Charlson Comorbidity Index score						<0.001
0	60,381 (92.4)	14,489 (90.0)	15,382 (92.6)	16,813 (93.1)	13,697 (94.0)	
1	3,639 (5.6)	1,067 (6.6)	932 (5.6)	967 (5.4)	673 (4.6)	
2	610 (0.9)	202 (1.3)	157 (0.9)	141 (0.8)	110 (0.7)	
3 or more	709 (1.1)	336 (2.1)	143 (0.9)	134 (0.7)	96 (0.7)	
**Surgeon characteristics**						
No. (%) of surgeons	2,536	2,178 (85.9)	182 (7.2)	118 (4.7)	58 (2.3)	
Age, mean (SD), years	43.7 (8.7)	43.9 (8.8)	43.9 (7.9)	42.3 (7.8)	41.4 (6.1)	0.201
No. (%) of Practice location						0.100
Urban	1,849 (72.9)	1,582 (72.6)	126 (69.2)	93 (78.8)	48 (82.8)	
Rural	687 (27.1)	596 (27.4)	56 (30.8)	25 (21.2)	10 (17.2)	

Table 1 also shows that patients with acute appendicitis were treated by 2,536 surgeons between 2007 and 2009, at a mean volume per surgeon of 25.8 (±53.2) cases. The ^χ2^ analyses indicate that the surgeons in the low-volume group were more likely to be female (*p*<0.001) and practice in non-teaching hospitals (*p*<0.001) than their counterparts in other volume groups. No significant relationships were found in the surgeon distributions in terms of mean age (*p* = 0.201) and practice location (*p* = 0.100) across caseload volume groups.

Table 2 presents the distributions of the rates of perforated appendicitis according to surgeon and patient characteristics. Global χ^2^ analyses revealed that there were significant differences in the rate of perforated appendicitis associated with surgeon practice location (*p*<0.001), teaching status of practice hospital (*p*<0.001), patient age (*p*<0.001), patients gender (*p*<0.001), and CCI (*p*<0.001).

**Table 2:** Distributions of perforated appendicitis by surgeon and patient characteristics and comorbidities (n = 65,339).

Variable	Perforated appendicitis		*p* value
	Yes, *n*(row %)	No, *n*(row %)	
**Surgeon characteristics**			
Surgeon age (years)			0.217
<41	711 (42.2)	20,794 (42.9)	
41∼50	6,758 (40.1)	19,326 (39.9)	
>50	2,984 (17.7)	8,366 (17.2)	
Surgeon gender			0.989
Male	16,284 (96.6)	46,848 (96.6)	
Female	569 (3.4)	1,638 (3.4)	
Practice location			<0.001
Urban	13,323 (79.1)	36,874 (76.1)	
Rural	3,530 (20.9)	11,612 (23.9)	
Teaching status			<0.001
Yes	15,660 (92.9)	43,162 (89.0)	
No	1,193 (7.1)	5,324 (11.0)	
**Patient characteristics**			
Patient age (years)			<0.001
≤19	3,452 (20.5)	10,320 (21.3)	
20∼39	4,443 (26.4)	20,877 (43.1)	
40∼59	5,024 (29.8)	12,477 (25.7)	
≥60	3,934 (23.3)	4,812 (9.9)	
Patient gender			<0.001
Male	9,883 (58.6)	25,526 (52.7)	
Female	6,970 (41.4)	22,960 (47.3)	
Charlson Comorbidity Index score			<0.001
0	14,612 (86.7)	45,769 (94.4)	
1	1,564 (9.3)	2,075 (4.3)	
2	307 (1.8)	303 (0.6)	
3 or more	370 (2.2)	339 (0.7)	

Table 3 provides the crude and covariate-adjusted odds ratio (OR) estimates of the likelihood of perforated appendicitis by surgeon acute appendicitis case volume and surgeon and patient characteristics. The ORs of perforated appendicitis for those patients treated by medium-volume, high-volume, and very-high-volume surgeons were 0.95 (95% CI = 0.90–0.99), 0.91 (95% CI = 0.86–0.95), and 0.70 (95% CI = 0.66–0.73), respectively, compared to patients treated by low-volume surgeons. After adjusting for surgeon practice location, and teaching status of practice hospital, and patient age, gender, CCI, and hospital acute appendicitis case volume, patients treated by low-volume surgeons had significantly higher rates of perforated appendicitis than those treated by medium-volume surgeons (OR = 1.09, reciprocal of 0.92, *p*<0.001), high-volume surgeons (OR = 1.16, *p*<0.001), or very-high-volume surgeons (OR = 1.54, *p*<0.001). In addition, it is also worth noting that higher rates of perforated appendicitis were detected among patients treated by surgeon practicing in the urban areas (adjusted OR = 1.09) and teaching hospitals (OR = 1.61).

**Table 3:** Crude and adjusted odds ratios for perforated appendicitis, by surgeon acute appendicitis case volume.

Variables	Crude odds ratio		Adjusted odds ratio	
	95% CI	P value	95% CI	P value
**Surgeon acute appendicitis case volume**				
≤65	1.00		1.00	
66∼120	0.95 (0.90–0.99)	0.031	0.92 (0.87–0.96)	<0.001
121∼190	0.91 (0.86–0.95)	<0.001	0.86 (0.82–0.91)	<0.001
≥191	0.70 (0.66–0.73)	<0.001	0.66 (0.62–0.70)	<0.001
**Surgeon characteristics**				
Practice location				
Urban	1.19 (1.14–1.24)	<0.001	1.09 (1.03–1.14)	<0.001
Rural	1.00		1.00	
Hospital teaching status				
Yes	1.62 (1.52–1.73)	<0.001	1.61 (1.49–1.73)	<0.001
No	1.00		1.00	
**Patient characteristics**				
Patient age (years)				
≤19	1.00		1.00	
20∼39	0.64 (0.61–0.67)	<0.001	0.65 (0.62–0.68)	<0.001
40∼59	1.20 (1.14–1.27)	<0.001	1.18 (1.12–1.24)	<0.001
≥60	2.44 (2.31–2.59)	<0.001	2.19 (2.06–2.33)	<0.001
Patient gender				
Male	1.28 (1.23–1.32)	<0.001	1.33 (1.28–1.38)	<0.001
Female	1.00		1.00	
Charlson Comorbidity Index score				
0	1.00		1.00	
1	2.36 (2.21–2.53)	<0.001	1.54 (1.43–1.66)	<0.001
2	3.17 (2.71–3.72)	<0.001	1.81 (1.53–2.13)	<0.001
3 or more	3.42 (2.95–3.95)	<0.001	2.01 (1.73–2.35)	<0.001
**Hospital appendicitis case volume**	1.00 (1.00–1.00)	<0.001	1.00 (1.00–1.00)	<0.001

## Discussion

To the best of our knowledge, this is the first study to use nationwide data to investigate the relationship between surgeon volume and the perforation rate of appendicitis. Our study demonstrated that low volume surgeons were more likely to have a greater number of perforated appendicitis.

Earlier studies have suggested that higher hospital volumes are associated with better outcomes for several surgical procedures [18], [19]. The proposed explanation for this inverse volume-outcome relationship is commonly known as the ‘practice makes perfect’ hypothesis. This hypothesis is based upon the rationale that a larger volume of patients provides clinicians with the opportunity to better hone their surgical skills and accumulate experience in operation management. Therefore, high-volume providers are more likely to achieve superior clinical performance on account of their greater experience, improved skills, and operational judgment.

In the case of acute appendicitis, the severity and complication rate are closely associated with the time interval between symptom onset and treatment [20], and the rupture rate is generally not the result of surgical technique related error but rather pre- and in-hospital delay. Therefore, high-volume surgeons who have more experience in dealing with patients with acute appendicitis may reduce unnecessary in-hospital delays and lead to better outcomes. A corollary to the “practice makes perfect” hypothesis then, is that low-volume surgeons with poor outcomes would be able to substantially reduce the rate of ruptured appendicitis by simply increasing their patient volumes or having the opportunity to practice under the guidance of more experienced high-volume surgeons.

A second hypothesis used to explain volume-outcome relationships involves ‘selective-referral’. This hypothesis suggests that patients are selectively referred to providers with superior outcomes. Thus, the best providers have the highest volumes on account of their prior record of superior outcomes. However, this hypothesis is unlikely to offer a valid explanation for this study. The progression of appendicitis is rapid, and most patients promptly receive treatment at nearby hospitals. Therefore, ‘selective-referral’ may not be a major factor contributing to the inverse relationship between the patient outcome sand surgeon volumes observed in this study.

The main strength of this study was its use of a nation-wide population-based dataset from Taiwan. Taiwan is a country with more than 23 million people on a 35.8 thousand square kilometer island, which launched a universal national health insurance (NHI) program in the end of 1995. Under a single-payer system operated by the Taiwanese government, the NHI program covered 98% of Taiwan’s population by 2009. The population-based dataset associated with this program offered the statistical power necessary to detect real differences. Furthermore, Taiwan is over 98% Han Chinese, and this study could therefore exclude the influence of race and insurance status from our analysis, both of which were previously demonstrated to be important factors influencing the rate of ruptured appendicitis [21], [22].

There are some limitations to this study. First, as pathologic confirmation was unavailable in this study, the definition of perforated appendicitis was dependent on ICD-9 codes sourced from an administrative database. These codes may be less accurate than data collected prospectively. For example, while ICD-9-CM code 540 is theoretically reserved exclusively for uncomplicated appendicitis, we cannot rule out the possibility that miscoding occurred. Furthermore, as coding is a skill itself, it is possible that lower volume surgeons would have been more likely to miscode more specific codes referring to complicated appendicitis (ICD-9-CM codes 540.0 and 540.1) as ICD-9-CM code 540. If this were the case, our results would be biased toward an underestimation of the effect size. Secondly, although this study has controlled for patient co-morbidities, the administrative database used by this study is extremely limited in its ability to account for the differences in the severity of appendicitis among patients. Therefore it is possible that the associations detected in this study were biased by the residual confounding of severity. Third, this study also lacked any data describing the rate of negative appendectomies which may have biased our results, as higher rates of negative appendectomies would have decreased the rate of perforated appendicitis [23]. Fourth, the dataset used in this study did not have any data on pre-hospital care (the time from the onset of symptoms to first seeking medical attention), advanced diagnostic testing, or the use of antibiotics. Therefore, these potentially confounding factors could not be considered in our analysis. Finally, the very small appendectomy caseloads seen among some surgeons in the very low-volume group may have prohibited their meaningful statistical comparison.

In conclusion, our study suggested that surgeon volume is an important factor with regard to the rate of ruptured appendicitis. Future investigations will need to be undertaken to determine a threshold of surgeon volume for best practice, and to identify the differences in clinical approach between high-volume surgeons with superior outcomes, and low-volume surgeons with inferior outcomes. The identification of such differences may be useful toward aiding low-volume surgeons with inferior outcomes to reduce the incidence of perforated appendicitis.

## References

[1] Addiss DG, Shaffer N, Fowler BS, Tauxe RV (1990) The epidemiology of appendicitis and appendectomy in the United States. Am J Epidemiol 132: 910–925. 2239906 10.1093/oxfordjournals.aje.a115734

[2] Williams GR (1983) Presidential Address: a history of appendicitis. With anecdotes illustrating its importance. Ann Surg 197: 495–506. 6342553 10.1097/00000658-198305000-00001 PMC1353017

[3] Nitecki S, Karmeli R, Sarr MG (1990) Appendiceal calculi and fecaliths as indications for appendectomy. Surg Gynecol Obstet171: 185–188. 2385810

[4] Williams NM, Jackson D, Everson NW, Johnstone JM (1998) Is the incidence of acute appendicitis really falling? Ann R Coll Surg Engl 80: 122–124. 9623378 PMC2502999

[5] Al-Omran M, Mamdani M, McLeod RS (2003) Epidemiologic features of acute appendicitis in Ontario, Canada. Can J Surg 46: 263–268. 12930102 PMC3211626

[6] Andersson RE (2007) The natural history and traditional management of appendicitis revisited: spontaneous resolution and predominance of prehospital perforations imply that a correct diagnosis is more important than an early diagnosis. World J Surg 31: 86–92. 17180556 10.1007/s00268-006-0056-y

[7] Balthazar EJ, Rofsky NM, Zucker R (1998) Appendicitis: the impact of computed tomography imaging on negative appendectomy and perforation rates. Am J Gastroenterol 93: 768–771. 9625125 10.1111/j.1572-0241.1998.222_a.x

[8] Fuchs JR, Schlamberg JS, Shortsleeve MJ, Schuler JG (2002) Impact of abdominal CT imaging on the management of appendicitis: an update. J Surg Res 106: 131–136. 12127818 10.1006/jsre.2002.6441

[9] Hannan EL, O'Donnell JF, Kilburn H Jr, Bernard HR, Yazici A (1989) Investigation of the relationship between volume and mortality for surgical procedures performed in New York State hospitals. JAMA 262: 503–510. 2491412

[10] Birkmeyer JD, Siewers AE, Finlayson EV, Stukel TA, Lucas FL, et al (2002) Hospital volume and surgical mortality in the United States. N Engl J Med 346: 1128–1137. 11948273 10.1056/NEJMsa012337

[11] Welke KF, Diggs BS, Karamlou T, Ungerleider RM (2009) Comparison of pediatric cardiac surgical mortality rates from national administrative data to contemporary clinical standards. Ann Thorac Surg 87: 216–222. 19101301 10.1016/j.athoracsur.2008.10.032

[12] Lien WC, Lee WC, Wang HP, Chen YC, Liu KL, et al (2011) Male gender is a risk factor for recurrent appendicitis following nonoperative treatment. World J Surg 35: 1636–1642. 21573957 10.1007/s00268-011-1132-5

[13] Paquette IM, Zuckerman R, Finlayson SR (2011) Perforated appendicitis among rural and urban patients: implications of access to care. Ann Surg 253: 534–538. 21209586 10.1097/SLA.0b013e3182096d68

[14] Lien YC, Huang MT, Lin HC (2007) Association between surgeon and hospital volume and in-hospital fatalities after lung cancer resections: the experience of an Asian country. Ann Thorac Surg 83: 1837–1843. 17462409 10.1016/j.athoracsur.2006.12.008

[15] Wen HC, Tang CH, Lin HC, Tsai CS, Chen CS, et al (2006) Association between surgeon and hospital volume in coronary artery bypass graft surgery outcomes: a population-based study. Ann Thorac Surg 81: 835–842. 16488681 10.1016/j.athoracsur.2005.09.031

[16] Ponsky TA, Huang ZJ, Kittle K, Eichelberger MR, Gilbert JC, et al (2004) Hospital- and patient-level characteristics and the risk of appendiceal rupture and negative appendectomy in children. JAMA 292: 1977–1982. 15507583 10.1001/jama.292.16.1977

[17] Ditillo MF, Dziura JD, Rabinovici R (2006) Is it safe to delay appendectomy in adults with acute appendicitis? Ann Surg 244: 656–660. 17060754 10.1097/01.sla.0000231726.53487.dd PMC1856602

[18] Lin HC, Xirasagar S, Lee HC, Chai CY (2006) Hospital volume and inpatient mortality after cancer-related gastrointestinal resections: the experience of an Asian country. Ann Surg Oncol 13: 1182–1188. 16897270 10.1245/s10434-006-9005-0

[19] Nallamothu BK, Saint S, Hofer TP, Vijan S, Eagle KA, et al (2005) Impact of patient risk on the hospital volume-outcome relationship in coronary artery bypass grafting. Arch Intern Med 165: 333–337. 15710799 10.1001/archinte.165.3.333

[20] Pittman-Waller VA, Myers JG, Stewart RM, Dent DL, Page CP, et al (2000) Appendicitis: why so complicated? Analysis of 5755 consecutive appendectomies. Am Surg 66: 548–554. 10888130

[21] Smink DS, Fishman SJ, Kleinman K, Finkelstein JA (2005) Effects of race, insurance status, and hospital volume on perforated appendicitis in children. Pediatrics 115: 920–925. 15805365 10.1542/peds.2004-1363

[22] Camp M, Chang DC, Zhang Y, Arnold M, Sharpe L, et al (2010) Provider density and health system facility factors and their relationship to rates of pediatric perforated appendicitis in US counties. Arch Surg 145: 1139–1144. 21173286 10.1001/archsurg.2010.271

[23] Hale DA, Molloy M, Pearl RH, Schutt DC, Jaques DP (1997) Appendectomy: a contemporary appraisal. Ann Surg 225: 252–261. 9060580 10.1097/00000658-199703000-00003 PMC1190674

